# SA Rugby Injury and Illness Surveillance and Prevention Project (SARIISPP): Super Rugby Unlocked and the Carling Currie Cup, Premiership Competition, Injury Surveillance Report, 2020/21

**DOI:** 10.17159/2078-516X/2021/v33i1a11849

**Published:** 2021-01-15

## Executive Summary

The outbreak of the Coronavirus pandemic (COVID19) resulted in the 2020 Super Rugby competition being suspended in March 2020, at completion of round 7 of the competition, and the 2020 Carling Currie Cup competition did not take place as originally scheduled. Seven South African Rugby franchises returned to official competition on 9 October 2020. The seven franchises first played in a 7-round 2020 Super Rugby Unlocked tournament, after which the winners of this part of the combined tournaments, the Vodacom Blue Bulls, were crowned as the Super Rugby Unlocked Champions. This was followed immediately by a 7-round 2020/21 Carling Currie Cup, with knockout play-off rounds. The points from the Super Rugby Unlocked tournament were carried over into the Carling Currie Cup. This culminated in the Vodacom Blue Bulls also being crowned as the Carling Currie Cup 2020/21 Champions after the final play-off match. As the Super Rugby Unlocked and Carling Currie Cup competitions included the same seven franchises, with no break afforded between competitions, this report presents the combined data from both tournaments. Although the structure of the 2020/21 season was unique, data analysis revealed no apparent reasons for these data to be treated differently to previous seasons’ data. As such the collective competition data is referred to as the ‘Carling Currie Cup’ 2020/21 throughout this report. Despite the disruptions to the 2020 season, and players potentially being unable to maintain the physical qualities necessary to protect themselves against injury, our analysis tells a positive story about how players were managed during these times, with injury rates being comparable to previous seasons’ injury rates, and within the expected season-to-season limits of variation for the Carling Currie Cup.

As part of the SA Rugby Injury and Illness Surveillance and Prevention Project (SARIISPP), the Carling Currie Cup 2020/21 Premiership Division Competition (the ‘Carling Currie Cup’) injury data were recorded throughout the tournament by the medical doctors and medical support staff of the respective teams. All seven teams were required to record every match and training injury that occurred in their team.

The injury rate of Time-Loss injuries for the Carling Currie Cup 2020/21 was 91 (76 to 106) injuries per 1000 player hours (mean and 95% confidence intervals), which is higher than the international rate of 81 (63 to 105) injuries per 1000 player hours [1], but within the expected limits of season-to-season variation for the Carling Currie Cup. This equates to 1.8 injuries per team per match.

The Cell C Sharks had the highest injury rate for Time-Loss injuries for the 2020/21 tournament and this was significantly higher than their 2014–2019 tournament average. Despite having the highest injury rate, the Cell C Sharks had the lowest average severity and thus experienced a low burden of injury. What this means, is that although they had a high number of injuries, they did not lose many days of training and match play due to these injuries. This finding is interesting to note as the Cell C Sharks ranked 2^nd^ in the tournament, and in previous years the teams who ranked in 1^st^ or 2^nd^ positions of the competition had significantly lower injury rates than those who ranked in last position [2]. The Vodacom Blue Bulls, who won both the Super Rugby Unlocked phase of the tournament and the Carling Currie Cup, had a moderate injury rate and average severity, resulting in them experiencing a moderate injury burden as a team. Conversely, the Xerox Golden Lions, who had the second lowest injury rate, had a high average severity of injury resulting in a high injury burden. This means, that although having a low number of injuries, these injuries resulted in many training and match days missed. Although teams may have a low injury rate, injuries of a high severity still represent a sizable burden to the team, resulting in many training and match days lost due to injury for that team. This highlights the importance of collecting severity data, and not simply injury rates on their own.

The average severity of Time-Loss injuries in the 2020/21 tournament was 18 days, which is lower than the 25 days reported in English Professional Rugby [3]. The median injury severity of all Time-Loss injuries was 4 days, with 25% of injuries lasting 1 day and 25% of injuries lasting 12 days or more due to injury.

Contusion/bruise injuries were the most common injury type in the 2020/21 tournament, with muscle (rupture/strain/tear) and sprained ligament injuries recording the second and third most common injury types, respectively. The head was the most commonly injured body location, with Concussions being the most common injury diagnosis for the fifth consecutive year. Open play accounted for the highest proportion of injuries in the 2020/21 tournament with *collision* being the secondary mechanism for 65% of the open play injuries. The injury rates for *being tackled* and *tackling* were comparable, however injuries from *tackling* carried a higher average severity than those from *being tackled.* The injury rate from the *ruck* was similar to both tackling and being tackled but had a lower average severity.

A total of 53 Time-Loss training injuries were sustained in the Carling Currie Cup 2020/21, meaning that 27% of all Time-Loss injuries, more than a quarter of all injuries, occurred in training activities. This equates to an incidence of 2.6 (1.9 to 3.2) injuries per 1000 training hours, which is comparable to the meta-analysis injury incidence of 3 (2 to 4) injuries per 1000 training hours [1]. The average severity of Time-Loss training injuries was 13 days, which is lower than the 20 days reported in the meta-analysis [1]. The majority (42%) of Time-Loss training injuries occurred in *Full-Contact Rugby Skills* training, followed by 32% in *Semi-Contact Rugby Skills* training.

In February 2020, the International Olympic Committee (IOC) published an updated consensus statement for methods of recording and reporting epidemiological data on injury and illness in sport [4]. The Carling Currie Cup 2020/21 report has aligned with this more recent consensus statement. In certain sections of this report, such as the subsequent injury section for example, additional details on injuries have been added to assist practitioners when they report these injuries. Details on these additions are described in the Definitions section of this report.[Fig f20-2078-516x-33-v33i1a11849]

**Figure f20-2078-516x-33-v33i1a11849:**
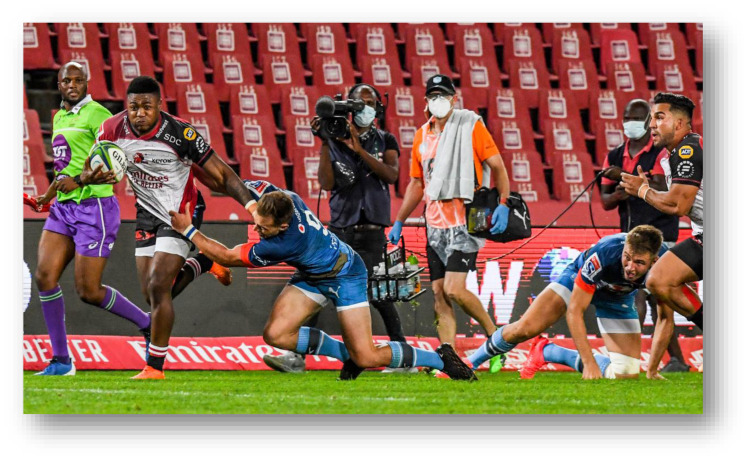


## Introduction

In 2014, as part of the SA Rugby Injury and Illness Surveillance and Prevention Project (SARIISPP), the South African Rugby Union (SA Rugby) implemented a new standardised injury surveillance format for the Carling Currie Cup Premiership Division Competition. This required the team doctor or medical support staff to electronically capture all relevant match and training injury data according to the standardised *BokSmart* injury surveillance data capture format, which is aligned with the IOC consensus statement for injury recording in sport [4], and for rugby union [5].

Injury surveillance is a critical step in the development, and testing of the efficacy and effectiveness, of injury prevention strategies. Injury surveillance captured in the correct format enables the comparison of injury rates between teams within the same tournament, tournament injuries over successive years, and with other rugby injury surveillance studies. Literature describing tournament injuries presents the injury numbers as a rate where the total number of injuries is divided by the total amount of time exposed to the risk of experiencing an injury. An injury rate is expressed as the number of events per 1000 player exposure hours. Match exposure hours are calculated as the number of matches played multiplied by the number of exposed players (15) and the match exposure time (80 mins). Training exposure hours are calculated as the average number of players present at training multiplied by the average time spent training each week; this is then summed to the get training exposure hours over the competition period. This normalised version of the injury number facilitates comparison between teams in 2020/21, previous tournaments and the international injury surveillance literature. Throughout this report the normalised injury rates have been provided to allow for comparison with other tournaments and the international literature, as discussed, but every effort has been made to present these rates on a ‘per team’ and ‘per match’ level for easier and more pragmatic interpretation.

Since 2016, the Carling Currie Cup doctors or medical support staff were asked to record the physical return to play date of the injured players, thereby allowing for the actual severity of the injury to be calculated. For those cases, where the player had not returned to play by the start of the following year, doctors or medical support staff were asked to provide an estimated return to play date. The severity of these injuries was then calculated using the estimated date provided, and not the actual date. Calculating the actual severity of most injuries adds substantial value to the report as it enables one to determine the burden of the teams’ injuries with greater accuracy. Injury burden is a combination of the injury rate and severity and is expressed as the number of days absent from training and matches per 1000 player hours. Throughout the report, in sections reporting on injury numbers and incidence only, the 2014 and 2015 season data are included, while sections reporting on injury severity and burden do not include the 2014 and 2015 season data, due to severity data only being captured from the 2016 season.

The Carling Currie Cup 2020/21 season saw the introduction of Time-Loss training injury and training exposure data being captured as a part of the SARIISPP. The addition of training exposure and injury information is a substantial addition to the SARIISPP, as it allows us to reflect a full competition injury picture. As this is the first season where training related data has been captured, this section is limited but will continue to grow and develop over the years.

It is important to note that a multitude of factors contribute to players’ injury risk and injury causing events. The medical, conditioning, coaching staff, and the players themselves are equally responsible for ensuring that players are medically, mentally, and physically fit to handle the demands of the competition. Additionally, each player has unique intrinsic and extrinsic injury risk factors, which are beyond the control of the team’s staff.

An inherent issue with most injury surveillance studies is that the teams’ medical doctors or medical support staff are exclusively responsible for entering their team’s injury data. As no audit process is done on the collection of these data, in many of these cases, the accuracy of the data is dependent on the compliance of the doctors or medical support staff. This potential limitation is present in most injury surveillance studies. To minimise this potential limitation, SARIISPP had a project coordinator who was in frequent contact with the doctors or medical support staff to ensure they were up to date with the data capturing.

The Carling Currie Cup 2020/21 semi-finals were contested between DHL Western Province vs. Cell C Sharks and Vodacom Blue Bulls vs. Xerox Golden Lions. The final was between Vodacom Blue Bulls vs. Cell C Sharks, with the Vodacom Blue Bulls eventually winning the tournament. The Vodacom Blue Bulls also won the Super Rugby Unlocked phase of the extended competition.[Fig f21-2078-516x-33-v33i1a11849]

**Figure f21-2078-516x-33-v33i1a11849:**
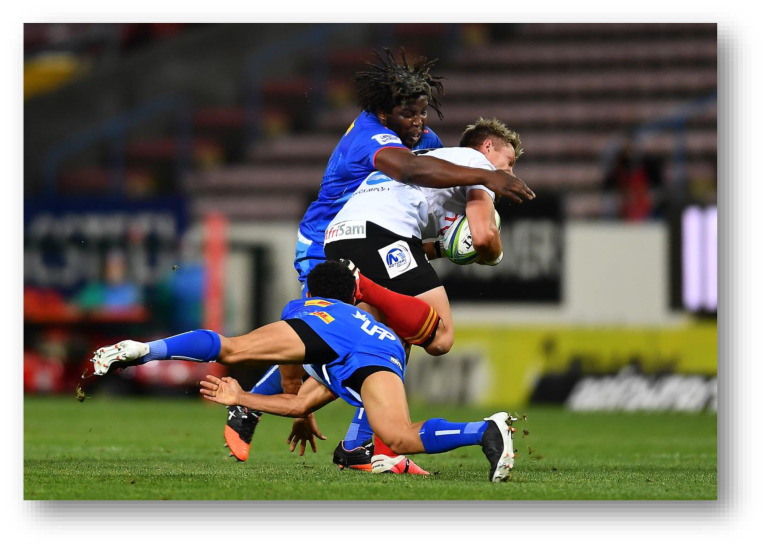


## Definitions

All definitions are originally based on the 2007 consensus statement for injury reporting in rugby union [5] and have since been realigned with the latest International Olympic Committee (IOC) consensus statement for methods of recording and reporting epidemiological data on injury and illness in sport [4].

### MEDICAL ATTENTION INJURY

All injuries that were seen by the teams’ doctor or medical support staff were classified as Medical Attention injuries, which are defined by the 2007 statement as an “*injury that results in a player receiving medical attention”* [5], and by the more recent IOC statement as *“a health problem that results in an athlete receiving medical attention”* [4].

### TIME-LOSS INJURY

Medical Attention injuries were further categorised as Time-Loss injuries, where appropriate, and defined by the 2007 statement as, “*an injury that results in a player being unable to take a full part in future rugby training or match play*” [5]. The IOC definition is, *“a health problem that results in a player being unable to complete the current or future training session or competition”* [4].

### INJURY RATE

For this report, an injury rate is the number of injuries expressed per 1000 player exposure hours. This method of expressing injury rate has been used in previous years’ reports of the Carling Currie Cup Premiership tournament and other international literature, and therefore makes comparisons easy. Moreover, the injury rate is expressed as a mean with 95% confidence intervals. A 95% confidence interval around a mean value indicates that there is a 95% chance (i.e., very high chance) that the true value falls within this range. In this report, we present the 95% confidence intervals assuming normal distribution of the data and use the approach of examining the overlap of the confidence intervals, to determine whether the injury incidences are significantly different; if the range of confidence interval values of two comparisons do not overlap, there is a strong chance (95%) that their injury rates are different from each other. We have opted for this method because it is easy to use, conservative and less likely to produce false positive results [6].

### MEDIAN (INTERQUARTILE RANGE)

When numbers are ordered from the lowest to highest, the median is the value which separates the higher half of the values from the lower half of the values. Simply put, it is the middle value of a list of ranked numbers. The interquartile range (IQR) describes the spread of the data. When rank ordered data are divided into quartiles the first and the third quartile represents the value under which 25% and 75% of the data points fall, respectively. As an example, a team may have a median injury severity of 32 days (IQR 7 to 40). This means that when the teams’ injury severities are rank ordered the mid-point or median of the injury severities is 32 days. Also 25% of their injuries result in 7 or less days absent from training and matches and 25% of their injuries result in 40 days or more absent from training and matches.

### META-ANALYSIS

A meta-analysis is a study using statistical methods to combine multiple scientific studies with varying levels of evidence on the same topic to determine overall defining patterns and results from the combined data. As such, it represents the highest level of scientific evidence available. The findings in this report are compared to that of the most recent meta-analysis for rugby union injuries at a senior professional level [1]. Although this was published in 2013, it remains the most comprehensive assessment of injuries associated with rugby.

### NEW, SUBSEQUENT AND RECURRENT INJURIES

In 2019, in the Carling Currie Cup Premiership Division Competition, a ‘*New Injury’* was defined as when a player sustained his first injury. Any injury that the *same* player sustained after this initial injury was defined as a *‘Subsequent Injury’.*

According to the more recent IOC statement, any subsequent injury to the same site and of the same type is referred to as a ‘*Recurrence’* if the index injury was fully recovered before reinjury, and as an *‘Exacerbation’* if the index injury was not yet fully recovered [4].

To provide more detail on the subsequent injuries for practitioners, we have further categorized the subsequent injuries in this report into one of four groups based on the OSICS classification diagnosis:

- Different site - Different type
- Different site - Same type
- Same site - Different type
- Same site - Same type

According to the 2007 Consensus Statement for rugby, any subsequent injury classified as ‘Same site - Same type’ was a *‘Recurrent injury’* [5].

### INJURY SEVERITY

The total severity of an injury is defined as *“the number of days that have elapsed from the date of injury to the date of the player’s return to full participation in team training and availability for match selection”* [4], [5]. For each year, at the time of injury the doctors or medical support staff were asked to estimate the severity of the injury based on their clinical assessment of the injured player. These estimations were made according to the severity groupings provided in the 2007 consensus statement; *Slight* (0–1 days lost), *Minimal* (2–3 days lost), *Mild* (4–7 days lost), *Moderate* (8–28 days lost), *Severe* (>28 days lost), *Career ending* and *Non-fatal catastrophic* [5]. To align with the latest IOC statement the injuries have been re-grouped to reflect the severity groupings *‘1–7 days’, ‘8–28 days’ and ‘>28 days’* [4].

The average severity represents the average number of days lost per injury when dividing the accumulated total number of days lost by the total number of injury events. For example, a team may have a total severity of 550 days absent, accumulated from 22 injuries. The average severity of the team’s injuries would therefore be 550/22, which equals, on average 25 days absent per injury.

### INJURY BURDEN

Injury burden is a combination of injury rate and severity. It is the injury rate multiplied by the average severity (number of days lost due to injury) and is expressed as the number of days absent per 1000 player hours. For example, a team who has an injury rate of 75 injuries per 1000 player exposure hours, and an average severity of 38 days lost per injury will have an injury burden of 2850 days absent per 1000 player hours (75 x 38).

### OPERATIONAL INJURY BURDEN

The operational burden is the expected number of days lost per injury per team for every match played over the tournament or season. The measure is an extrapolation of injury rates and severities over a season and includes the most severe injuries together with the least severe injuries in its estimation. For example, if a team has an operational injury burden of 2 days, it means that based on their injury rates and average severity, on average, 2 days absence can be expected from every match injury the team sustains.

## MATCH INJURIES

### Injured players

During the ‘Carling Currie Cup’ 2020/21, 99 players sustained a total of 146 Time-Loss injuries. Since we cannot account for all players who entered or left the match day squads during the tournament, either by injury replacement or otherwise, the following assumption was made: a total of 161 players were exposed to playing rugby matches in the tournament (7 teams x 23 players per match-day squad). Of these 161 players, 61% sustained a match injury at some stage in the tournament (Figure 1a). The proportion of players who experienced one Time-Loss injury decreased from 2019 to 2020/21, while the proportion of players who experienced 2 or 3 injuries increased from 2019 to 2020/21 (Figure 1b). Further analyses will focus on the absolute injury numbers, regardless of the number of players who sustained them.

### Overall Injury Rate

Only the number of Time-Loss injuries, those which resulted in the player missing more than one training session or match, were considered for further analysis, because these injuries are more comparable between different teams, tournaments and with the published scientific literature [5].

**Figure 2:**
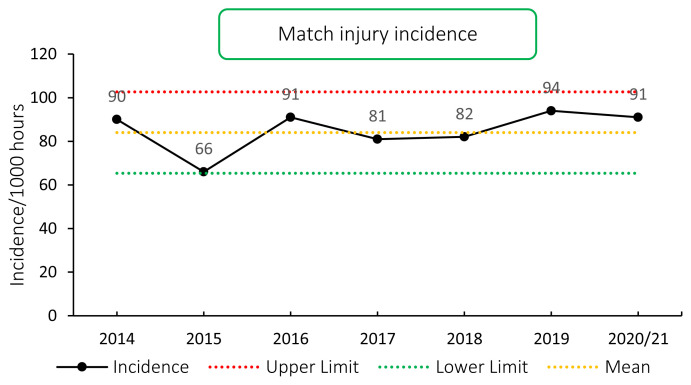
Injury incidence of Time-Loss match injuries over the surveillance period with mean ± standard deviations shown.

The overall match injury incidence for the Carling Currie Cup 2020/21 was 91 (76 to 106) injuries per 1000 player exposure hours. This is higher than the incidence of the meta-analysis (81 injuries per 1000 player hours, 63 to 105) [1], but is within the expected limits of season-to-season variation for the Carling Currie Cup (Figure 2). An injury incidence of 91 injuries per 1000 player hours equates to 1.8 injuries per team per match.

**Figure 3:**
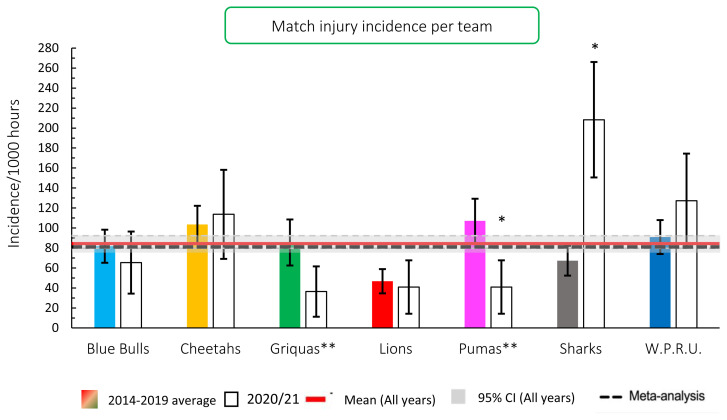
Injury incidence for Time-Loss injuries experienced by each team in the Carling Currie Cup 2020/21 in comparison to their 2014–2019 averaged injury rate. (**) Average injury rates for Pumas 2015 – 2019 and Griquas for 2015, 2016 and 2018, 2019. Asterisk (*) indicates that a team’s 2020/21 injury rate is significantly different to their 2014–2019 averaged injury rate.

When comparing the team’s 2014–2019 averaged tournament injury incidence to their 2020/21 injury incidence, the Phakisa Pumas experienced a significantly lower incidence in 2020/21 in comparison to their 2015–2019 tournament average (Figure 3; they never played in this division in 2014). The Cell C Sharks had a significantly higher incidence in 2020/21 in comparison to their 2014–2019 tournament average.

It remains interesting to note that the combined mean incidence and 95% CI for all teams for all years, 84 (79 to 90) injuries per 1000 player hours is similar to the summary of international data described in the meta-analysis of 81 (63 to 105) injuries per 1000 player hours [1].

### Influence of the COVID19 suspension

**Figure 4:**
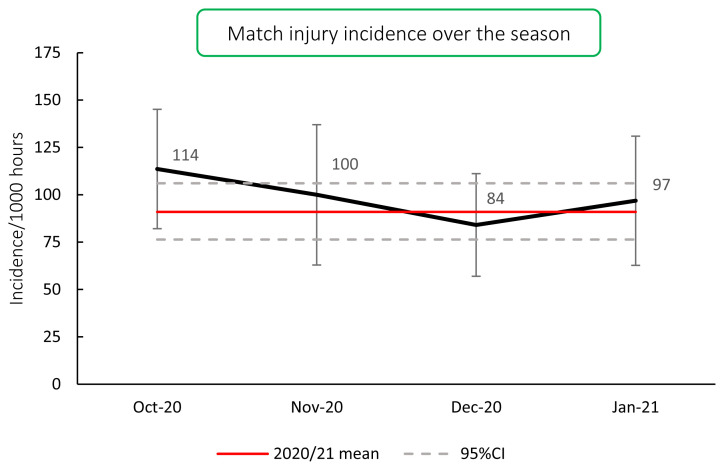
Match injury incidence per month of the 2020/21 Currie Cup season.

Despite the outbreak of COVID19 and suspension of regular team training and match play, when players returned to competition, the overall 2020/21 season injury incidence [91 (76 to 106) injuries per 1000 player hours] was not significantly different to the 2014–2019 tournament mean [84 (79 to 90) injuries per 1000 player hours (Figure 3)]. Looking at the Time-Loss match injury incidence over the course of the 2020/21 season shows that there was no month in the season where the match injury incidence was significantly different to the 2020/21 mean injury incidence or to any other month in the season (Figure 4).

### Overall Severity

**Figure 5:**
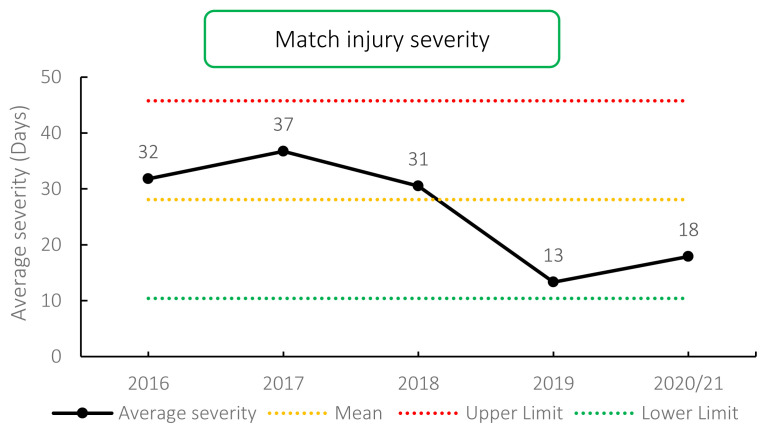
Mean severity of Time-Loss match injuries over the surveillance period with mean ± standard deviations shown.

The average severity of match injuries for the Carling Currie Cup 2020/21 was 18 days, which is lower than the average for the surveillance period 2016–2020/21 (28 days) but was within the expected limits of season-to-season variation (Figure 5). The median severity was 4 days (IQR 1 to 12). This means that the half-way mark of the injury severities was 4 days, with 25% of all Time-Loss injuries lasting for 1 day and 25% lasting 12 days or longer.

For each year, at the time of injury the doctors or medical support staff were asked to estimate the severity of the injury, based on their clinical assessment of the injured player. A *‘Slight’* injury refers to 0–1 days, *‘Minimal’* is 2–3 days, *‘Mild’* is 4–7 days, *‘Moderate’* is 8–28 days and *‘Severe’* is >28 days off rugby training and/or match play. These severity groupings are according to the original 2007 consensus statement for rugby union [5].

To align these data with the latest IOC statement the injuries have been re-grouped to reflect the severity groupings *‘1–7 days’, ‘8–28 days’ and ‘>28 days’* [4].

**Figure 6:**
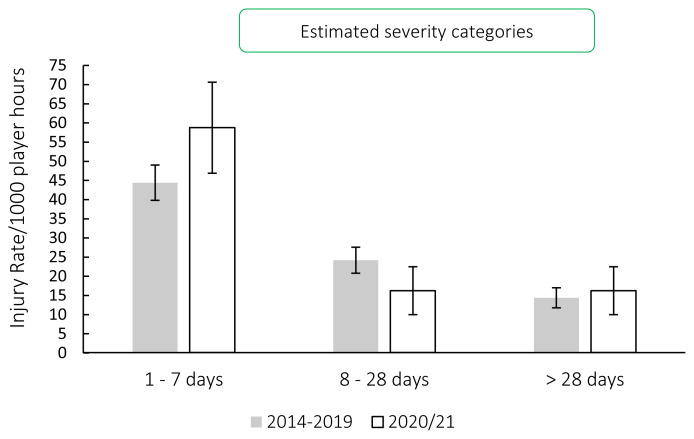
The estimated severity category injury rates for the Carling Currie Cup 2020/21 in comparison to the averaged injury rates for the 2014–2019 estimated severity categories using the definitions from the latest IOC statement[4].

Figure 6 compares the estimated injury severity rates for the Carling Currie Cup 2020/21 tournament to the 2014–2019 tournament averaged rate. There were no significantly different injury severity rates in 2020/21 in comparison to previous years (Figure 6).

Table 1 describes the actual severity of each teams’ Time-Loss injuries for the Carling Currie Cup 2020/21. The Vodacom Blue Bulls have again been used as a worked example to explain the Table. The Vodacom Blue Bulls sustained 1.3 injuries per match, meaning that for every 0.8 matches played they sustained one injury. In total, the Vodacom Blue Bulls lost 321 training and match days due to injury. This equates to an average of 20 training and match days lost for every injury sustained. The burden of the team’s injuries equates to 1235 days lost per 1000 player hours. Translating this to an operational burden per match, it shows that the Vodacom Blue Bulls lost 24.7 days per injury per match over the season. The median injury severity for the Vodacom Blue Bulls was 9 days (IQR 5 to 28). This means that when severities of the Vodacom Blue Bulls Time-Loss injuries were rank ordered, the midpoint of the severities was 9 days off from rugby, with 25% of their injuries lasting equal to or less than 5 days off and 25% of their injuries lasting equal to or longer than 28 days off (Table 1).[Fig f22-2078-516x-33-v33i1a11849]

**Figure f22-2078-516x-33-v33i1a11849:**
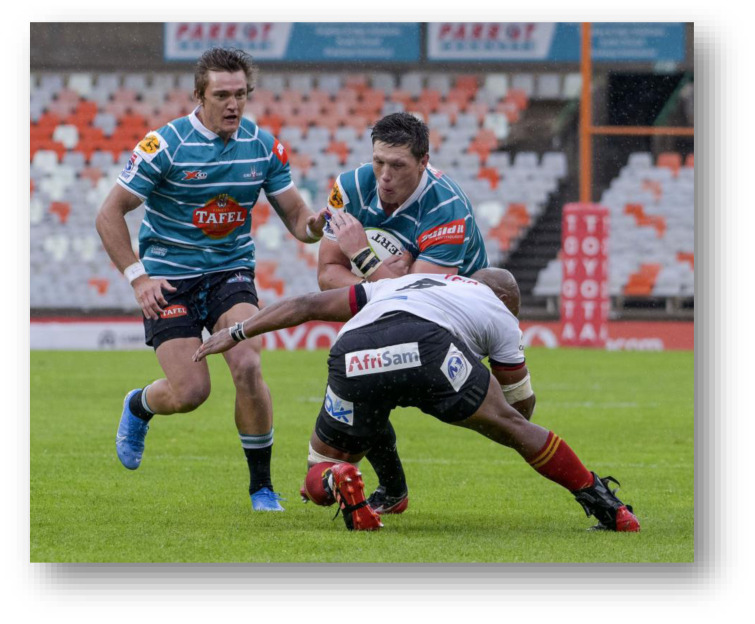


**Figure 7:**
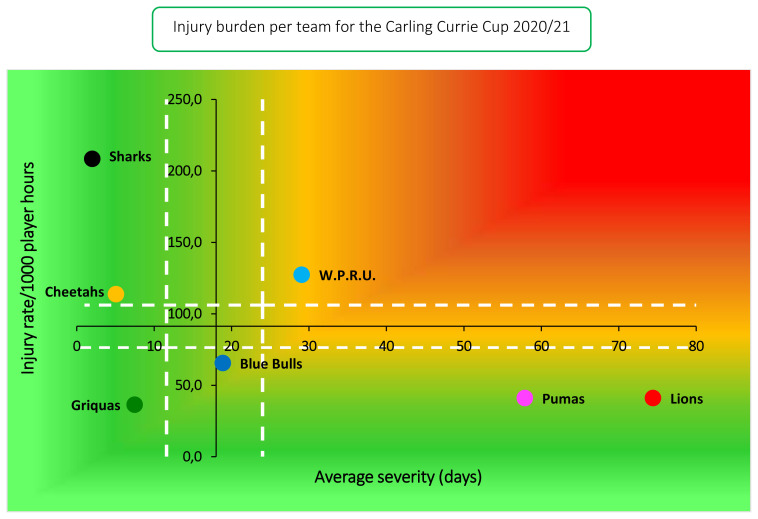
Injury rate plotted against the average severity of Time-Loss injuries for each participating team in the Carling Currie Cup 2020/21. The Y-axis Average Injury Rate is expressed as the tournament average (±95% CI) and X-axis Average Severity is expressed as the average (±95% CI) of the individual injury severities in the tournament.

The Cell C Sharks had the highest rate of Time-Loss injuries, but these were of low severity. Conversely, the Xerox Golden Lions had a low injury rate, but their injuries were of high severity (Figure 7). Teams who fall in the green zone (below average and 95%CI), will generally not be impacted as much by their injury burden, regardless of whether their injury rate or average severity is relatively high. As soon as the combination of rate and severity moves into the orange (close to average) and/or red zone (above average and 95% CI), the impact on team performance and player availability becomes more problematic.

The data in this report is aligned with the most recent IOC consensus statement [4] and is further presented such that it facilitates comparison with previous season reports and the meta-analysis [1]. Table 2 presents the Carling Currie Cup 2020/21 injury data in the format recommended by the most recent IOC consensus statement. This provides an overview of the 2020/21 season’s data in this format, with the data explored in more detail throughout the report.

### New, Subsequent and Recurrent Injuries

Overall, the injury rate for New injuries for the Carling Currie Cup 2020/21 was 62 (50 to 74) injuries per 1000 player hours, which is lower than the meta-analysis [1] rate of 78 (74 to 83) injuries per 1000 player hours. The average severity for New injuries in the Carling Currie Cup 2020/21 was 8 (5 to 11) days, which is lower than the average severity reported in the meta-analysis [1] of 20 (15 to 24) days.

**Figure 8:**
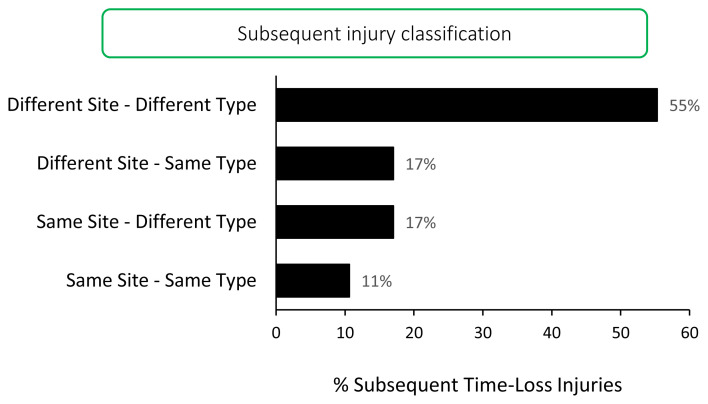
Classification of subsequent injuries for the Carling Currie Cup 2020/21. Data expressed as a % of subsequent Time-Loss injuries.

There were 32 players who sustained more than one injury in the Carling Currie Cup 2020/21. The majority (n = 26, 55%) of the subsequent injuries were at a different anatomical site and of a different type when compared to the first injury. These ‘*different site – different type*’ injuries together with the ‘*different site – same type*’ and ‘*same site – different type*’ injuries, would be classified as subsequent new injuries. (Figure 8).

A subsequent recurrent injury was any subsequent injury classified as ‘same site – same type’, which refers to the same location and same tissue type involved as the original index injury. There were five subsequent recurrent injuries in the Carling Currie Cup 2020/21. The overall injury rate for subsequent recurrent injuries was 3 (0.4 to 5.9) injuries per 1000 player hours, which is significantly lower than the meta-analysis rate of 11 (10 to 12) injuries per 1000 player hours [1]. When comparing the new and subsequent recurrent injuries across the Carling Currie Cup 2016 – 2020/21 tournaments, there was a decrease in the proportion of new injuries and increase in the proportion of subsequent *recurrent* injuries in 2020/21 in comparison to previous seasons (Table 3).

### Injury Type

Contusion/bruise injuries (21%, n = 31) were the most common Time-Loss injuries recorded in the Carling Currie Cup 2020/21, with muscle (rupture/strain/tear) injuries comprising the second highest proportion (17%, n = 25). The median severity for contusion/bruise injuries was 2 days with 25% of injuries resulting in 1 day absent from training and matches, and 25% of injuries resulting in 4 or more days absent from training and matches (Table 4).[Fig f23-2078-516x-33-v33i1a11849]

**Figure f23-2078-516x-33-v33i1a11849:**
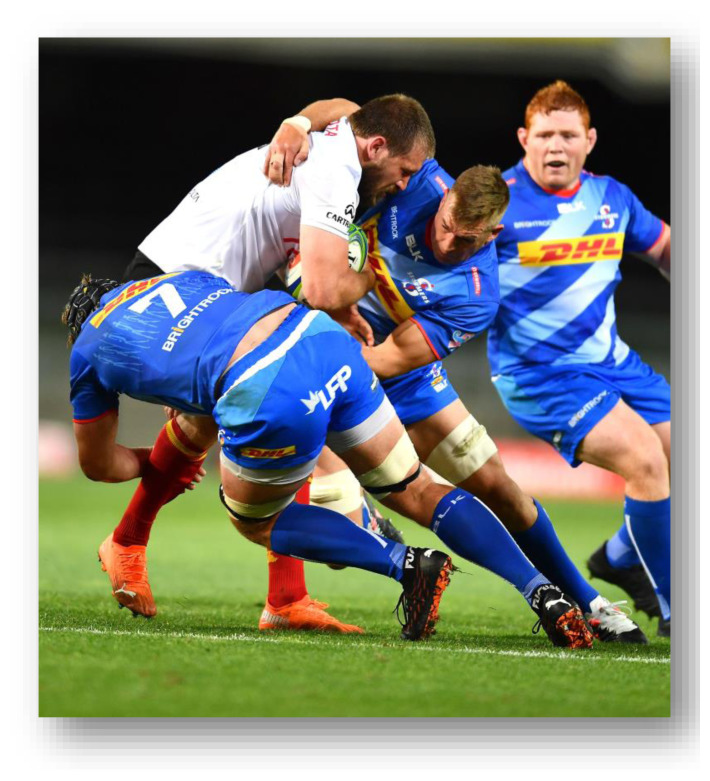


**Figure 9:**
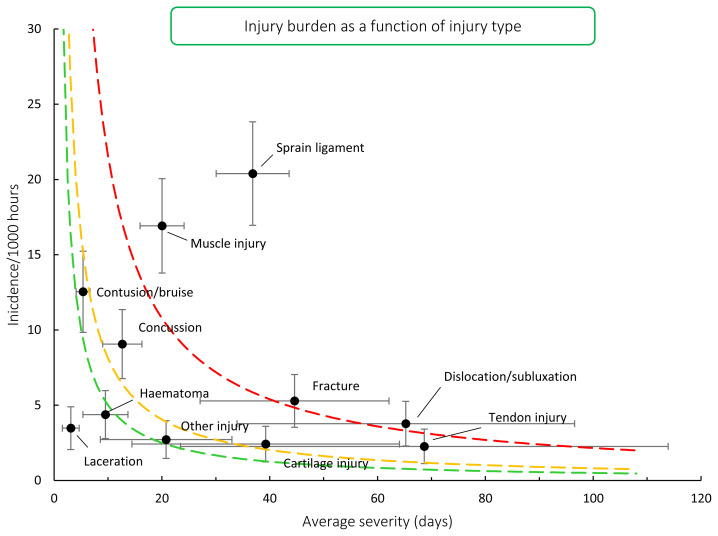
Injury burden as a function of injury type for the seasons 2016 – 2020/21. The y-axis represents incidence (number of injuries per 1000 hours), and x-axis represents the average severity (days absence) per injury type. Green line: values to the left and below represent those under the 25^th^ burden percentile; these are low-risk injuries. Orange line: values to the left and below represent those under the 50^th^ burden percentile; these include the low-medium risk injuries. Red line: values to the left and below represent those under the 75^th^ burden percentile; these include the medium-high risk injuries. Values to the right and above the red line are the most high-risk types of injuries, and impact players and teams the most.

When the injury types from 2016 – 2020/21 were combined, ligament sprain injuries had the highest burden for teams, as they had both a high injury rate and average severity of injury. While not as high as ligament injuries, muscle injuries also had a high injury rate and average severity (Figure 9). Therefore, these two injury types still dominate, and impact teams more than the other injury types do.

For further comparison, the Carling Currie Cup 2020/21 Time-Loss injury types were grouped in a similar way to the meta-analysis of international studies [1]. With these groupings, aligned to the meta-analysis, the most common Time-Loss injury types in the Carling Currie Cup 2020/21 were joint (non-bone)/ligament injuries (comprised of dislocation/subluxation and sprain/ligament injuries).

The injury rate of 7 (3 to 11) injuries per 1000 player hours for the central nervous system during the Carling Currie Cup 2020/21, was comparable to the rate of “central/peripheral” system injuries of the meta-analysis, 8 (4 to 15) injuries per 1000 player hours [1].

The injury rate for muscle/tendon injuries (comprised of muscle rupture/strain/tear, tendon injury/rupture and tendinopathy injuries) was 18 (12 to 25) injuries per 1000 player hours. This was lower than the same type of injury grouping in the meta-analysis, which had a rate of 40 (21 to 76) injuries per 1000 player hours, albeit not significantly different [1]. The average severity for muscle/tendon injuries of 19 (13 to 28) days, in the Carling Currie Cup 2020/21 was similar to the average severity of 15 (5 to 24) days reported in the meta-analysis [1].

In contrast, joint (non-bone)/ligament injuries (comprised of dislocation/subluxation and sprain/ligament injuries) were comparable: 19 (13 to 26) injuries per 1000 player hours in the present study, compared to the 24 (18 to 65) injuries per 1000 player hours in the meta-analysis [1]. The average severity of joint (non-bone)/ligament injuries in the Carling Currie Cup 2020/21 was 31 (27 to 54) days, which is comparable to the average severity for the same types of injuries reported at 29 (19 to 39) days in the meta-analysis[1].

### Injury Diagnosis [7]

The most frequent OSICS classification diagnosis^7^ in the Carling Currie Cup 2020/21 was HNCX Concussion. Concussions have remained the most frequently diagnosed injury for the past 5 seasons (Table 5).

## Concussions

**Figure 10:**
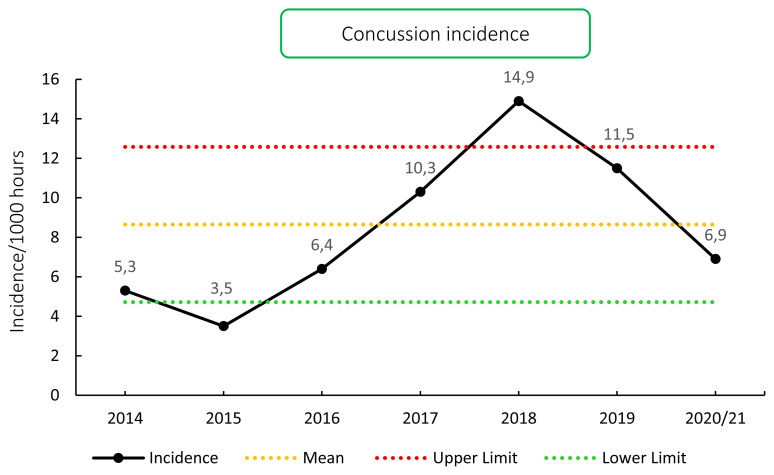
Incidence of concussion over the surveillance period with mean ± standard deviations shown.

Concussions contributed to 8% (n = 11) of all Time-Loss injuries for the Carling Currie Cup 2020/21, which equates to an incidence of 6.9 (2.8 – 10.9) concussions per 1000 hours. Concussion incidence has trended downwards since 2018, and an incidence of 6.9 is slightly lower than the mean for the surveillance period but falls within the expected limits of season-to-season variation, for the Carling Currie Cup (Figure 10). The average severity of concussions reported in the 2020/21 tournament was 10 days (IQR 5 – 18 days). The current South African Rugby concussion regulations do not normally allow for adult players to return within less than 12 days of the concussive event. As this competition takes place at the professional level and is a World Rugby approved tournament, Advanced Care protocols are implemented and carried out by the medical practitioner that could potentially allow a player to return-to-play in less than 12 days.

Advanced care clinical settings are defined in the World Rugby and SARU’s Concussion Guideline documents:

- World Rugby Concussion Guideline document - https://playerwelfare.worldrugby.org/
- SARU’s Concussion Guideline documents (When can a player safely return-to-play following a concussion) www.boksmart.com/concussion

**Figure 11:**
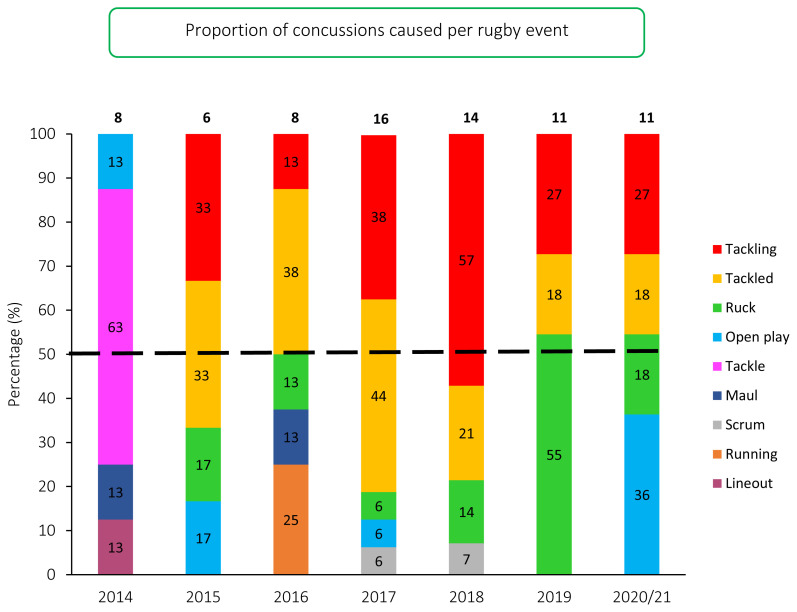
Proportion of concussions caused by the different injury events from 2014 to 2020/21. (The number above each bar represents the total number of concussions for that year. Tackle data captured separately as tackling and tackled from 2015 onwards).

The proportion of concussions caused from Tackling (27%) and from being Tackled (18%) in the Carling Currie Cup in 2020/21 was identical to the 2019 season. In the 2019 season the majority of concussions were caused in the *Ruck* (55%), with the proportion of concussions occurring in the *Ruck* decreasing to 18% in the 2020/21 season (Figure 11). The majority of the concussions in the 2020/21 season came from *Open play* (36%), which were all caused by *Collisions.*

**Figure 12:**
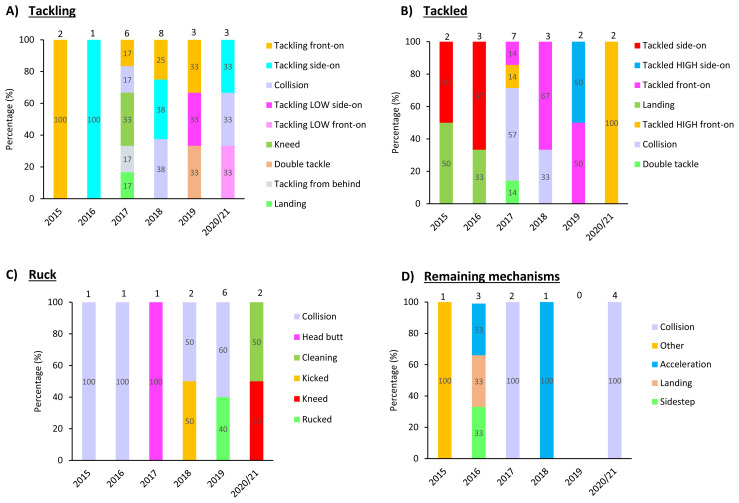
Proportion of concussions caused by A) Tackling, B) Tackled, C) Ruck and D) Remaining concussion mechanisms from 2015 to 2020/21. (The number above each bar represents the total number of concussions for that event for that year.)

The mechanisms contributing to concussions in *Tackling, Tackled, Ruck* and the remaining concussion causing events from 2015 – 2020/21 have been presented in Figures 12 A – D. Data have only been presented from 2015 onwards as *Tackle* related data were not captured separately for the *Tackler* and *Ball Carrier* in 2014.

### Region of Injury

The most frequently injured body location during the Carling Currie Cup 2020/21 was the Head (16%, n = 23), followed by the Knee (15%, n = 15). Injuries to the head comprised of concussion (n = 11), bruising (n = 2) and lacerations (n = 10). The average burden of head injuries in 2020/21 was 84 days absent per 1000 player hours. The median severity of head injuries in 2020/21 was 5 days absent, with 25% of head injuries resulting in 2 or less days lost from training and matches, and 25% of all head injuries resulting in 9 or more days lost from training and matches (Table 6).[Fig f24-2078-516x-33-v33i1a11849]

**Figure f24-2078-516x-33-v33i1a11849:**
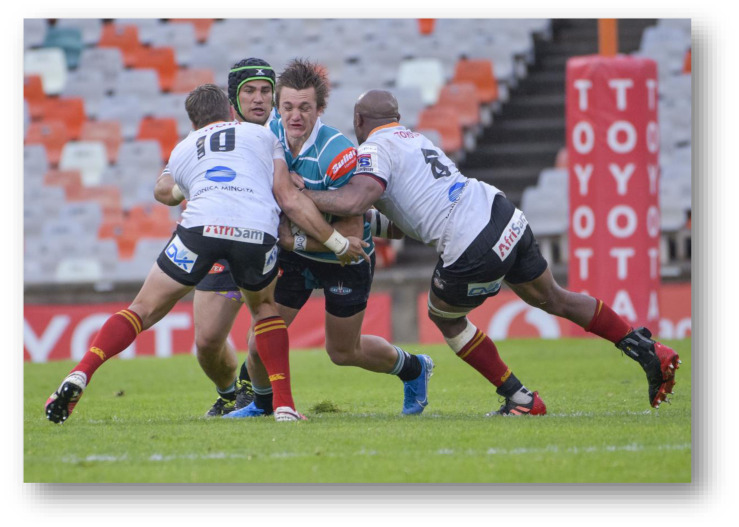


When looking at the movement of the most common body locations for the Carling Currie Cup over the past five seasons, the head and knee have remained the first and second most injured body locations respectively from 2017–2020/21. While the incidence of injuries to the head and knee in the 2020/21 season are similar to that in 2019, the average severity of injuries to the knee has increased (Table 7).

**Figure 13:**
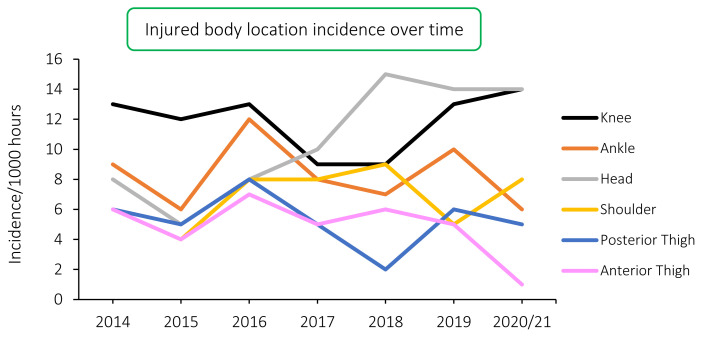
Incidence of the most common injury locations over the surveillance period.

When looking at the injury incidence of the most common injury locations over the surveillance period, the most noticeable trend is the initial increase in incidence of injuries to the head from 2014–2018, followed by a stabilization or levelling off thereafter. With the majority of injuries to the head comprising of concussions, this trend reflects a similar pattern to that seen in the concussion section above, albeit that the concussion rates decreased over the last two years. A variable trend in injury incidence is depicted in the other injury locations (Figure 13).

**Figure 14:**
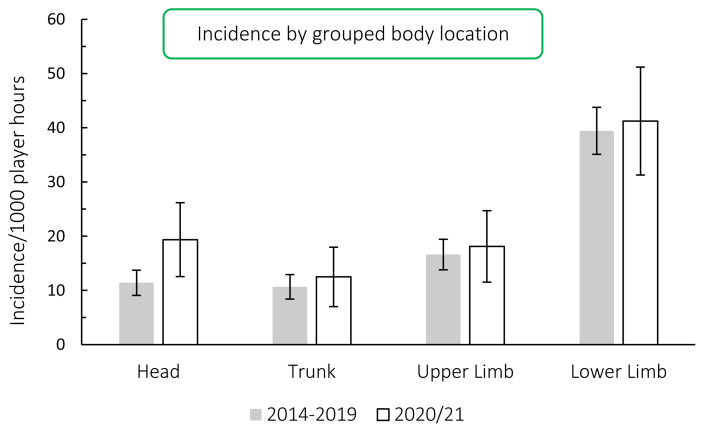
Injury incidence by grouped body location for the Carling Currie Cup 2020/21 in comparison to the averaged 2014–2019 injury rates.

When anatomical body locations were grouped for comparison with the data from the meta-analysis [1], the lower limb recorded the highest injury rate for the Carling Currie Cup 2020/21, with an injury rate of 41 (31 to 51) injuries per 1000 player hours, which was similar to that of the meta-analysis [1] at 47 (28 to 46) injuries per 1000 player hours (Figure 14). The injury rates for all grouped body locations in the Carling Currie Cup 2020/21 were comparable to their averaged 2014–2019 injury rates. The lower limb had the highest average severity of 29 days per injury, followed by the upper limb at 16 days per injury.

### Injury Event

In the Carling Currie Cup 2020/21, *open play* accounted for the highest proportion (33%, n = 48) of injury causing events, with *collision* being the secondary mechanism for 65% of these injuries. The injury rates for *being tackled* (13 (8 to 19) injuries per 1000 player hours) and *tackling* (12 (7 to 17) injuries per 1000 player hours) were comparable to each other and to the meta-analysis [1]; with injury rates for *being tackled* at 12 (5 to 19) injuries per 1000 player hours and *tackling* at 19 (12 to 29) injuries per 1000 player hours, in the meta-analysis. The *ruck* also added to this with 13 (8 to 19) injuries per 1000 player hours: a similar rate to both ball carrier and tackler roles, and comparable to the meta-analysis rate for the *ruck* of 17 (11 to 26) injuries per 1000 player hours [1].

**Figure 15:**
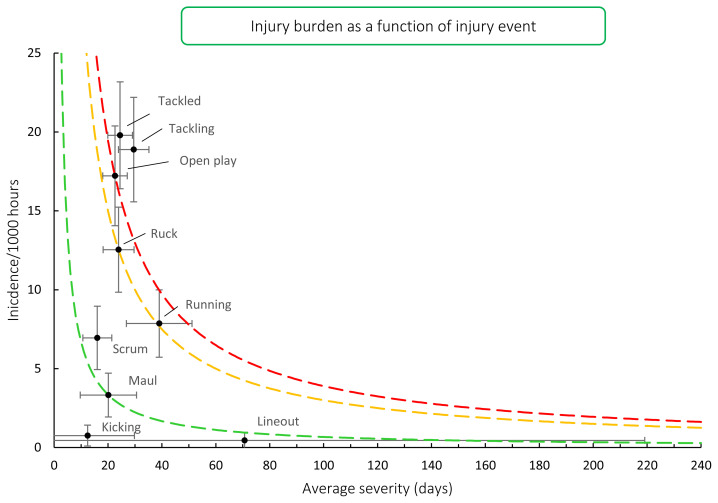
Injury burden as a function of injury event for the seasons 2016 – 2020/21. The y-axis represents incidence (number per 1000 hours), and x-axis represents average severity (days absence). Green line: values to the left and below represent those under the 25^th^ burden percentile; these are low-risk injuries. Orange line: values to the left and below represent those under the 50^th^ burden percentile; these include the low-medium risk injuries. Red line: values to the left and below represent those under the 75^th^ burden percentile; these include the medium-high risk injuries. Values to the right and above the red line are the most high-risk types of injuries, and impact players and teams the most.

Combining injury types from 2016 – 2020/21 showed that injuries from *tackling* carry the highest burden for teams, followed closely by injuries from *being tackled*, as they both present with a high injury rate and a relatively higher average severity of injury. Open play followed closely behind these two events (Figure 15).[Fig f25-2078-516x-33-v33i1a11849]

**Figure f25-2078-516x-33-v33i1a11849:**
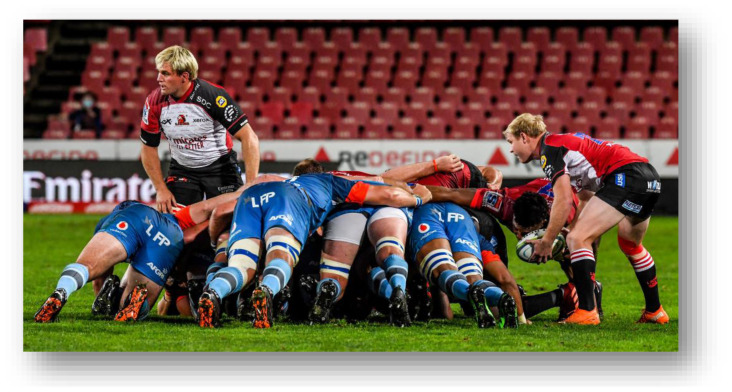


**Figure 16:**
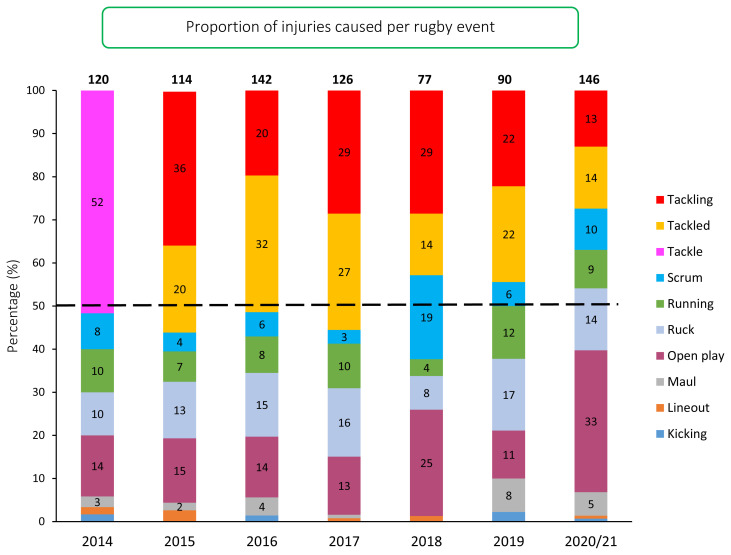
Proportion of injuries caused by the different injury events from 2014 to 2020/21. (The number above each bar represents the total number of injuries for that year. Tackle data captured separately as tackling and tackled from 2015 onwards).

Over the past four seasons the proportion of injuries caused by *Tackling* has decreased. The 13% of injuries in the 2020/21 season, was the lowest proportion recorded for *Tackling* over the surveillance period. A Chi^2^ test revealed that any changes in the proportion of injury causing events observed over the surveillance period were not statistically significant. The proportion of injuries sustained in *Open play* (33%) in the 2020/21 season was the highest recorded over the surveillance period (Figure 16).

### Venue

**Figure 17:**
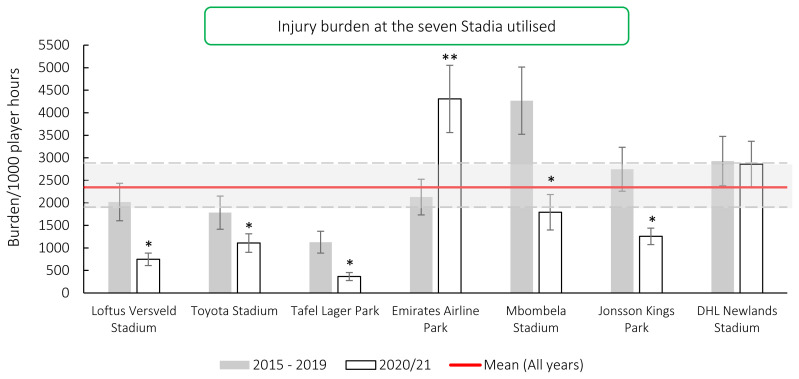
Injury burden/1000 player hours of Time-Loss injuries at the seven utilised stadia in the Carling Currie Cup 2020/21 in comparison to their averaged 2015–2019 injury burden. * Stadia’s injury burden was significantly lower in 2020/21 than their 2015–2019 average. **Stadium injury burden was significantly higher in 2020/21 than its 2015–2019 average.

Matches were played at seven stadia during the tournament. The following Stadia’s injury burden was significantly lower in 2020/21 than their 2015–2019 average: Mbombela Stadium, Jonsson Kings Park, Tafel Lager Park, Loftus Versfeld and Toyota Stadium. Emirates Airline Park’s injury burden was significantly higher in 2020/21 than its 2015–2019 average (Figure 17).

Combining all season’s data highlighted that Mbombela Stadium recorded the highest injury burden overall, with its injury burden being significantly higher than Jonsson Kings Park, Toyota Stadium, Loftus Versveld, Tafel Lager Park, and the grouped average injury burden from 2015–2020/21 (Table 9). Table 9 rank orders the Stadia from highest to lowest in terms of injury burden between 2015–2020/21.[Fig f26-2078-516x-33-v33i1a11849]

**Figure f26-2078-516x-33-v33i1a11849:**
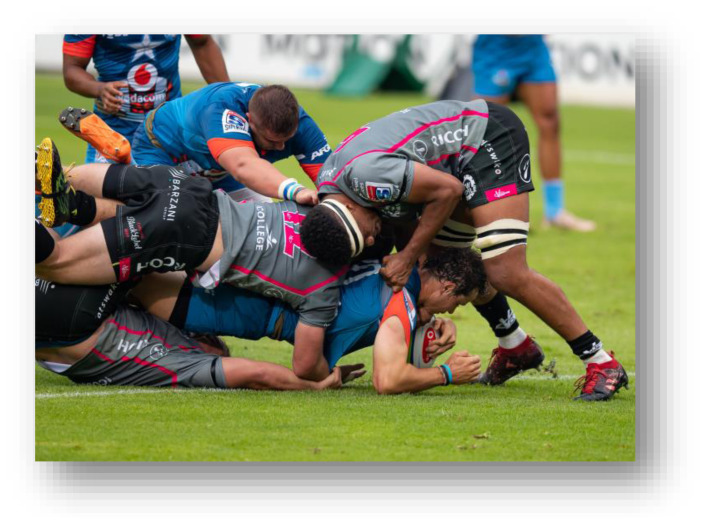


**Figure 18:**
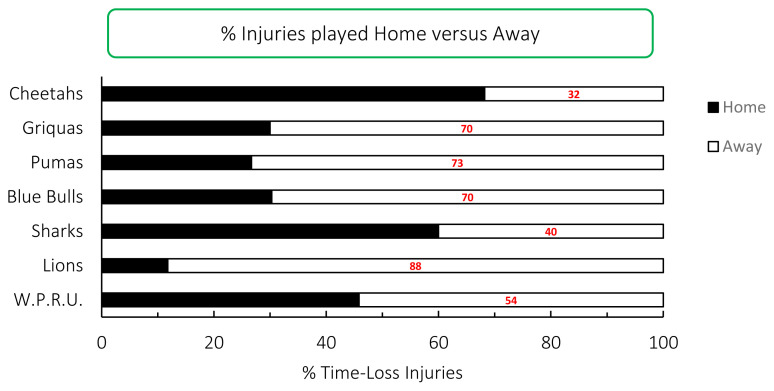
Proportion of injuries sustained playing at home and away venues for the Carling Currie Cup 2020/21.

Across all teams, and although not significant, playing at home [38 (28 to 47) injuries per 1000 player hours) had a lower injury rate than playing away [54 (42 to 59)] injuries per 1000 player hours) in the Carling Currie Cup 2020/21 tournament. The Cell C Sharks and Toyota Free State Cheetahs sustained more injuries playing at home than away, while all other teams sustained more injuries when playing away (Figure 18).

## TRAINING INJURIES

**Figure 19:**
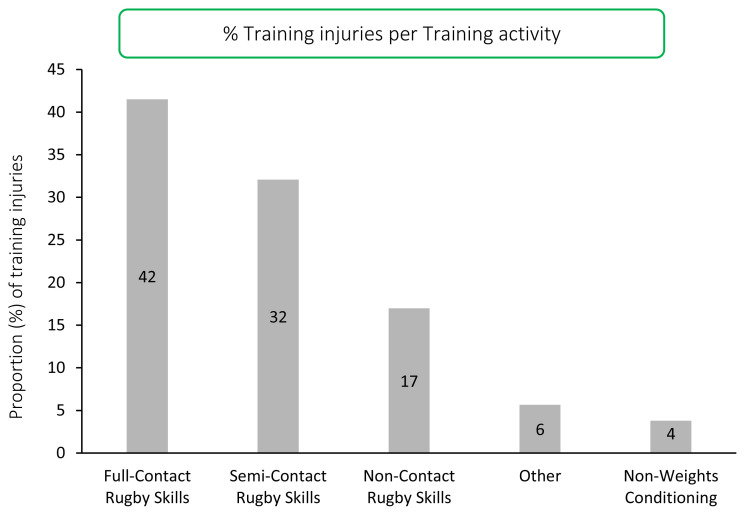
Proportion of Time-Loss training injuries sustained per training activity during the Carling Currie Cup 2020/21.

There was a total of 53 time-loss training injuries sustained in the Carling Currie Cup 2020/21, which equates to an incidence of 2.6 (1.9 to 3.2) injuries per 1000 training hours and is comparable to the meta-analysis injury incidence of 3 (2 to 4) injuries per 1000 training hours [1]. These contributed to more than a quarter (27%) of all injuries sustained by the Carling Currie Cup squads over the 2020/2021 rugby season. The average severity of training injury was 13 days, with a median severity (IQR) of 6 (2 to 17) days absent. An average severity of 13 days lost per training injury is slightly lower than the meta-analysis of 20 days [1]. The majority of training injuries occurred in *Full-Contact Rugby Skills* training (42%) and *Semi-Contact Rugby Skills* training (32%) (Figure 19).[Fig f27-2078-516x-33-v33i1a11849]

**Figure f27-2078-516x-33-v33i1a11849:**
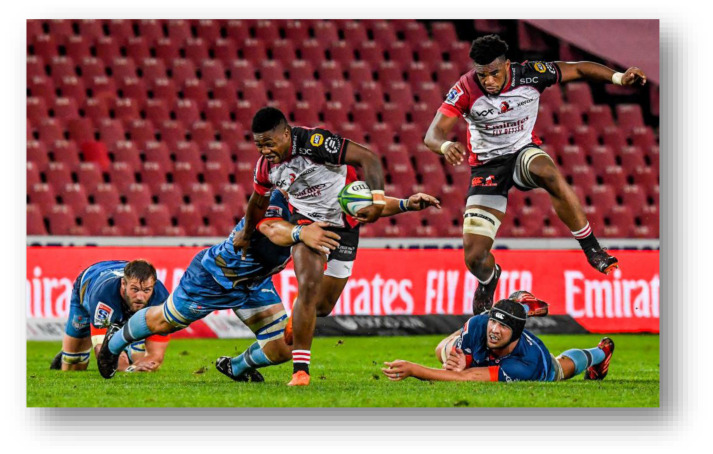


The most common injury type sustained in all training activities was *Muscle Injuries*, with *Ligament Sprain Injuries* sustained in *Full-Contact Rugby Skills* training carrying the highest average severity at 39 days (Table 10).

The knee was the most injured body location in training accounting for 15% (n = 8) of all Time-Loss training injuries during the Carling Currie Cup 2020/21 (Table 11).[Fig f28-2078-516x-33-v33i1a11849]

**Figure f28-2078-516x-33-v33i1a11849:**
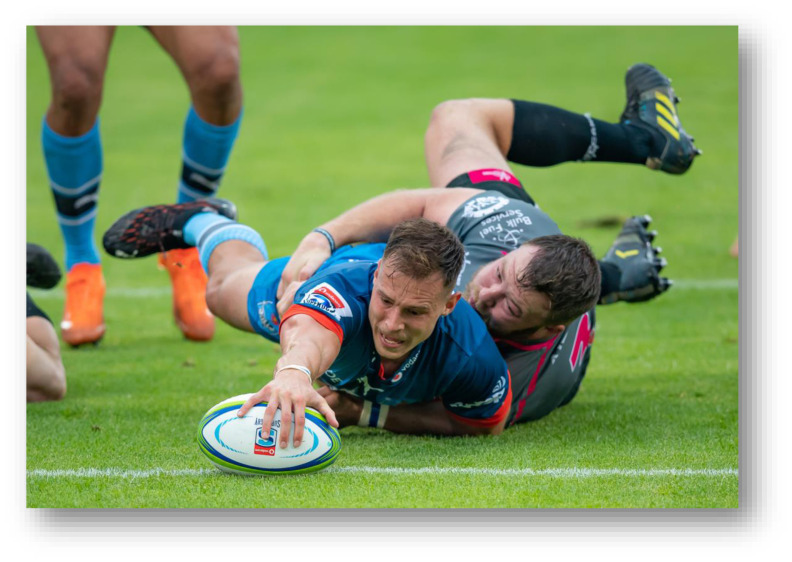


## Figures and Tables

**Figure 1a:**
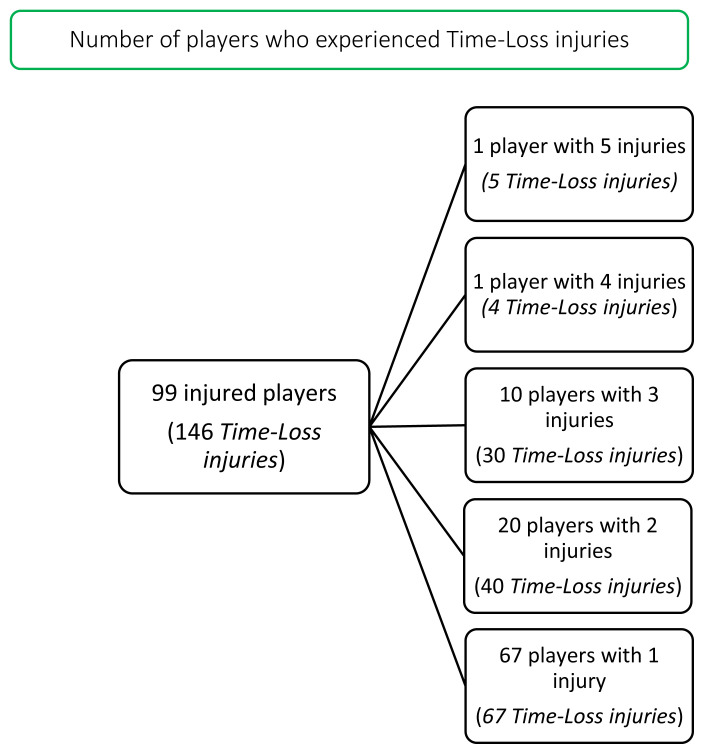
The number of players who experienced Time-Loss injuries during the Carling Currie Cup 2020/21.

**Figure 1b:**
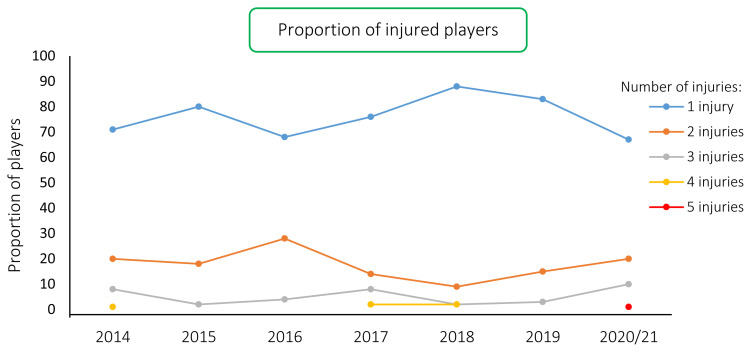
Proportion of injured players experiencing 1 or more injuries in the Carling Currie Cup tournaments from 2014–2020/21.

**Table 1:** Severity (days), Injury Burden (days absent/1000 player hours) and Operational Burden (days absent/injury/match) of Time-Loss injuries for each participating team in the Carling Currie Cup 2020/21.

	Team Injuries/match	Team matches/injury	Total Severity	Average Severity	Injury Burden	Operational Injury Burden	Median Severity (IQR)
Vodacom Blue Bulls	1.3	0.8	321	19	1235	24.7	9 *(5 to 28)*
Toyota Free State Cheetahs	2.3	0.4	127	5	577	11.5	3 *(2 to 4)*
Xerox Golden Lions	0.8	1.2	670	74	3045	60.9	7 *(6 to 135)*
Phakisa Pumas	0.8	1.2	521	58	2368	47.4	51 *(12 to 95)*
Cell C Sharks	4.2	0.2	101	2	421	8.4	1 *(1 to 2)*
DHL Western Province	2.5	0.4	814	29	3700	73.8	15 *(5 to 25)*
Tafel Lager Griquas	0.7	1.4	60	8	273	5.5	8 *(5 to 9)*

***Overall***	***1.8***	***0.5***	***2614***	***18***	***1634***	***33***	***4 (1 to 12)***

**Table 2:** The Carling Currie Cup 2020/21 injuries grouped according to the IOC recommended categories of Tissue and Pathology types for injuries.

Tissue	Injuries	Incidence	Median time-loss	Burden

*Pathology*	n	Injuries per 1000 hours (95%CI)	Days (95%CI)	Days per 1000 hours (95%CI)
***Muscle/Tendon***	***58***	***36.3 (27 to 46)***	***3 (4 to 10)***	***416.0 (322 to 538)***
Muscle Contusion	30	18.8 (12 to 25)	2 (2 to 4)	81.0 (57 to 116)
Muscle Injury	27	16.9 (11 to 23)	6 (6 to 18)	326.0 (224 to 475)
Tendinopathy	1	0.6 (0 to 2)	9	9 (1 to 64)
***Ligament/Joint capsule***	***42***	***26.3 (18 to 34)***	***4.5 (17 to 52)***	***1449.0 (1071 to 1961)***
Joint Sprain	21	13.1 (8 to 19)	2 (1 to 31)	336.0 (219 to 515)
Ligament Sprain	21	13.1 (8 to 19)	16 (22 to 84)	1113.0 (726 to 1707)
***Nervous***	***13***	***8.1 (4 to 13)***	***9 (6 to 11)***	***111.0 (64 to 191)***
Brain/Spinal cord injury	11	6.9 (3 to 11)	9 (7 to 12)	108.0 (60 to 195)
Peripheral nerve injury	2	1.3 (0 to 3)	1.5 (1 to 2)	3.0 (1 to 12)
***Superficial tissues/skin***	***11***	***6.9 (3 to 11)***	***2 (1 to 4)***	***24.0 (13 to 43)***
Laceration	11	6.9 (3 to 11)	2 (1 to 4)	24.0 (13 to 43)
***Bone***	***10***	***6.3 (2 to 10)***	***1.5 (0 to 50)***	***220.0 (118 to 409)***
Fracture	10	6.3 (2 to 10)	1.5 (0 to 50)	220.0 (118 to 409)
***Cartilage/Synovium/Bursa***	***8***	***5.0 (2 to 8)***	***28 (12 to 81)***	***372.0 (186 to 744)***
Synovitis/Capsulitis	2	1.3 (0 to 3)	21.5 (9 to 34)	43.0 (11 to 172)
Cartilage Injury	6	3.8 (1 to 7)	39.5 (11 to 99)	329.0 (148 to 732)
***Non-specific***	***2***	***1.3 (0 to 3)***	***8.5 (0 to 23)***	***17.0 (4 to 68)***
***Internal Organ***	***2***	***1.3 (0 to 3)***	***2.5 (2 to 3)***	***5.0 (1 to 20)***

***Overall***	***146***	***91.3 (76 to 106)***	***4 (12 to 24)***	***1633 (1330 to 2005)***

Where n = 1, median time-loss reflects the total time-loss days.

**Table 3:** Proportion (%) of new versus subsequent recurrent injuries for the Carling Currie Cup 2016 – 2020/21 tournaments.

	2016	2017	2018	2019	2020/21
New injuries	74	74	86	83	67
Subsequent Recurrent injuries	2.8	3.2	2.6	2.2	3.4

**Table 4:** Injury rate, Severity and Burden of the most common injury types in the Carling Currie Cup 2020/21.

Injury Type	Injury Rate *(95% CI)*	Total Severity	Average Severity	Burden *(95% CI)*	Median (IQR)
Contusion/Bruise	19 (12 to 25)	81	3	51 (32 to 80)	2 (1 to 4)
Muscle (Rupture/Strain/Tear)	17 (11 to 23)	326	12	204 (126 to 328)	6 (2 to 20)
Sprained Ligament	13 (8 to 19)	1113	53	696 (405 to 1195)	16 (3 to 84)
Lacerations	7 (3 to 11)	24	2	15 (7 to 32)	2 (1 to 3)
Central Nervous System	7 (3 to 11)	108	10	68 (32 to 143)	9 (6 to 12)

***Overall***	***91 (76 to 106)***	***2614***	***18***	***1633 (1330 to 2005)***	***4 (1 to12)***

**Table 5:** The movement of the most common OSICS classification diagnoses over the past five seasons^[7]^.

			%	Number	Incidence	Average Severity
2016		HN1 Concussion	7	10	6 (2–10)	14
		KL3 Knee medial collateral ligstr/tear/rupture	6	9	6 (2–10)	23
		TM1 Hamstring strain/tear	6	8	5 (2–9)	11
2017		HNCX Concussion	13	16	10 (5–15)	15
		SJAX Acromioclavicular jt sprain	10	12	8 (3–12)	25
2018		HNCX Concussion	18	14	15 (7–23)	14
		TMQX Quadricep strain	5	4	4 (0–8)	18
2019		HNCX Concussion	12	11	12 (5–18)	9
		AJSX Ankle syndesmosis sprain	5	5	5 (1–10)	14
2020/21		HNCX Concussion	8	11	7 (3–11)	10
		THV Quadriceps haematoma	4	6	4 (1–7)	4
		MCL Knee strain	3	5	3 (0.5–6)	42

**Table 6:** Injury rate, Severity and Burden of the most common injury types in the Carling Currie Cup 2020/21.

Injury Type	Injury Rate *(95% CI)*	Total Severity	Average Severity	Average Burden	Median (IQR)
Head	14 *(9 to 20)*	135	6	84 *(50 to 141)*	5 *(2 to 9)*
Knee	14 *(8 to 19)*	1260	57	788 *(464 to 1336)*	29 *(5 to 83)*
Thigh Injuries	9 *(5 to 14)*	132	9	83 *(43 to 156)*	2 *(2 to 6)*
Shoulder	8 *(4 to 13)*	292	22	183 *(92 to 363)*	6 *(2 to 28)*
Ankle	6 *(2 to 10)*	190	19	119 *(54 to 260)*	6 *(1 to 13)*

***OVERALL***	***91 (76 to 106)***	***2614***	***18***	***1633 (1330 to 2005)***	***4 (1 to 12)***

**Table 7:** The movement of the most injured body locations over the past five seasons.

			%	Number	Incidence	Average Severity
2016		Knee	14	20	13 (7–18)	49
		Ankle	13	18	12 (6–17)	51
		Head	9	13	8 (4–13)	11
		Shoulder	8	12	8 (3–12)	41
		Anterior thigh	8	12	8 (3–12)	49
2017		Head	13	16	10 (5–15)	15
		Knee	11	14	9 (4–14)	63
		Shoulder	10	12	8 (3–12)	67
		Ankle	10	12	8 (3–12)	87
		A/C Joint	10	12	8 (3–12)	25
2018		Head	18	14	15 (7–23)	18
		Knee	10	8	9 (3–14)	44
		Shoulder	10	8	9 (3–14)	38
		Ankle	9	7	7 (2–13)	65
		Anterior thigh	8	6	6 (1–12)	6
2019		Head	14	13	14 (6 – 21)	8
		Knee	13	12	13 (5 – 20)	13
		Ankle	11	10	10 (4 – 17)	9
		Lower limb posterior	7	6	6 (1 – 11)	3
		Posterior thigh	7	6	6 (1 – 11)	9
2020/21		Head	16	23	14 (9 to 20)	6
		Knee	15	22	14 (8 to 19)	57
		Thigh	10	15	9 (5 to 14)	9
		Shoulder	9	13	8 (4 to 13)	22
		Ankle	7	10	6 (2 to 10)	19

**Table 8:** Injury rate, Severity and Burden of the injury events in the Carling Currie Cup 2020/21

Injury Event	Injury Rate *(95% CI)*	Total Severity	Average Severity	Average Burden	Median (IQR)
Open play	30 *(22 to 38)*	475	10	297 *(208 to 425)*	2 *(1 to 5)*
Tackled (Ball Carrier)	13 *(8 to 19)*	317	15	198 *(115 to 340)*	6 *(2 to 16)*
Ruck	13 (*8* to 19)	489	23	306 *(178 to 525)*	5 *(2 to 20)*
Tackle (Tackler)	12 *(7 to 17)*	557	29	348 *(197 to 615)*	6 *(2 to 51)*
Scrum	9 *(4 to 13)*	176	13	110 *(57 to 213)*	8 *(1 to 16)*
Running	8 *(4 to 13)*	531	41	332 *(167 to 660)*	6 *(4 to 37)*
Maul	5 *(2 to 8)*	19	2	12 *(5 to 29)*	2 *(1 to 3)*
Lineout	1 *(0 to 2)*	28	28	18 *(1 to 209)*	28
Kicking	1 *(0 to 2)*	22	22	14 *(1 to 164)*	22

***OVERALL***	***91 (76 to 106)***	***2614***	***18***	***1633 (1330 to 2005)***	***4 (1 to 12)***

**Table 9:** Injury burden/1000 hours of Time-Loss injuries at the seven Stadia utilized in the Carling Currie Cup combined data from 2015 to 2020/21.

Stadium	Burden (95%CI)
Mbombela Stadium	**3719 (3044 to 4543)** *
DHL Newlands Stadium	2917 (2379 to 3575)
Emirates Airline Park	2602 (2126 to 3185)
Jonsson Kings Park	2439 (2026 to 2936) *
Toyota Stadium	1635 (1305 to 2047) *
Loftus Versveld	1446 (1155 to 1810) *
Tafel Lager Park	959 (749 to 1229) *

Grouped Average	2182 (1769 to 2693) *

* Mbombela Stadium’s injury burden is significantly higher than Jonsson Kings Park, Toyota Stadium, Loftus Versveld, Tafel Lager Park and grouped average

**Table 10:** Number, average, and median severity of training injuries sustained during the Carling Currie Cup 2020/21 season according to type of training activity involved.

	Number	Average severity (days)	Median severity (days)
***Full-Contact Rugby Skills***	***22***	***17***	***9***

Muscle Injury	7	17	9
Ligament Sprain	4	39	40
Laceration	3	2	2
Concussion	2	11	11
Muscle Contusion	2	2	2
Synovitis/Capsulitis	1	17	17
Organ Trauma (eye)	1	1	1
Fracture	1	34	34
Non-specific	1	2	2

***Semi-Contact Rugby Skills***	***17***	***13***	***6***

Muscle Injury	10	12	6
Synovitis/Capsulitis	2	7	7
Joint Sprain	2	13	13
Cartilage Injury	1	38	38
Laceration	1	7	7
Non-specific	1	6	6

***Non-Contact Rugby Skills***	***9***	***7***	***4***

Muscle Injury	6	4	3
Synovitis/Capsulitis	1	1	1
Fracture	1	4	4
Ligament Sprain	1	33	33

***Conditioning Non-weights***	***2***	***14***	***14***

Muscle Injury	2	14	14

***Other***	***3***	***5***	***2***

Nerve Injury	1	4	4
Tendinopathy	1	2	2
Synovitis/Capsulitis	1	9	9

**Overall**	**53**	**13**	**6**

**Table 11:** Number, average, and median severity of training injuries sustained per body location, during the Carling Currie Cup 20120/21.

	Number	Average severity (days)	Median severity (days)
Knee	8	16	13
Lower Leg Injuries	7	15	6
Head	7	5	2
Hamstring	5	9	6
Chest	5	15	7
Neck	3	8	2
Ankle	3	27	27
Thigh Injuries	2	1	1
Pelvis	2	31	3
Groin	2	6	6
Lumbar spine	2	2	2
Hand	1	4	4
Face	1	2	2
Shoulder	1	68	68
Elbow	1	7	7
Thoracic spine	1	1	1
Wrist	1	5	5
Quadriceps	1	17	17

**Overall**	**53**	**13**	**6**

## Footnotes

7 Orchard, J. *Orchard Sports Injury Classification System (OSICS)* 2019; Available from: https://www.johnorchard.com/osics-downloads.html.
